# The correlation between urinary iodine levels and gallstone risk: elevated iodine intake linked to gallstone occurrence

**DOI:** 10.3389/fnut.2024.1412814

**Published:** 2024-07-24

**Authors:** Yunfan Li, Minchen Wang, Wenyi Du, Liuyao Qi, Xiaopeng Liu, Xin Fan

**Affiliations:** ^1^Department of General Surgery, Affiliated Hospital of Jiangsu University, Zhenjiang, China; ^2^Department of General Surgery, The Affiliated Wuxi People’s Hospital of Nanjing Medical University, Wuxi, China

**Keywords:** trace elements, iodine intake, NHANES, urinary iodine, gallstones

## Abstract

**Background:**

Essential trace elements are vital for human growth and development. Nevertheless, excessive intake can pose risks. As of yet, no research has looked at the possibility of a relationship between the prevalence of gallstones and urinary concentrations of nickel, molybdenum, and iodine.

**Objectives:**

The purpose of this study was to examine the correlation between urinary levels of iodine, molybdenum, and nickel and the occurrence of gallstones in a U.S. population and to verify whether excessive iodine intake is associated with the occurrence of gallstones.

**Methods:**

Data from 2,734 participants that were gathered between 2017 and 2020 were examined. Employing inductively coupled plasma mass spectrometry (ICP-MS), the levels of nickel (Ni), iodine (I), and molybdenum (Mo) in the urine were determined. Gallstones presence was determined using a standardized questionnaire. Restricted cubic spline analysis, subgroup analysis, and logistic regression analysis were used to evaluate the relationship between the occurrence of gallstones and urinary essential trace elements.

**Results:**

The logistic regression analysis indicated an increased risk of gallstone development in Quartiles 2, Quartiles 3, and Quartiles 4 groups in comparison to the Quartiles 1 group, based on urinary iodine levels (OR = 1.69, 95% CI: 1.11–2.56; OR = 1.68, 95% CI: 1.10–2.55; OR = 1.65, 95% CI: 1.09–2.51). Urinary iodine levels were nonlinearly positively linked with the development of gallstones, according to restricted cubic spline analysis (*P*-Nonlinear = 0.032). Subgroup analyses showed that high levels of urinary iodine were associated with a high risk of gallstones in different populations, and were more pronounced in adults aged 60 years and older, in women, with a BMI ≥ 25, and in diabetic patients.

**Conclusion:**

Our research revealed a correlation between an increased risk of gallstones and increasing urinary iodine levels. Urinary iodine levels serve as indicators of the body’s iodine status, thus suggesting that excessive iodine intake may be linked to an elevated risk of gallstone formation.

## 1 Introduction

The primary cause of gallstones, a common digestive ailment marked by the production of stones in the bile ducts or gallbladder, is unusually high cholesterol levels in the bile. About 10–20% of persons worldwide suffer from gallstones, which has a substantial financial impact on individuals (1–4). Even though 80% of individuals with gallstones may not display any signs, the disease can progress from carriers with no symptoms to individuals with symptoms and complicated issues such as acute pancreatitis, cholangitis, and acute cholecystitis if prompt treatment is not obtained (5, 6). Obesity is recognized as an important risk factor for the development of gallstones (1). Literature shows that obesity is strongly associated with complications such as cardiovascular disease, type 2 diabetes mellitus, malignant tumors, asthma, osteoarthritis, chronic back pain, obstructive sleep apnea, nonalcoholic fatty liver disease, and gallbladder disease (7). In addition, previous studies have shown that the occurrence of gallstones is strongly associated with age, gender (female), pregnancy, hypertension, diabetes, and hyperlipidemia (1, 8–10). Also, among different ethnicities, the prevalence of gallstones is higher in Hispanic populations in the Americas and South America (1). As for the income level of the country, the prevalence of gallstones is also higher in low-income countries and upper-middle-income countries (11).

The human body requires around 20 elements, including both metallic and non-metallic elements, to maintain normal physiological functions. Among these essential elements, there are certain trace elements that are vital to the human body, despite being present in very small quantities. Examples of these essential trace elements include iodine molybdenum, and nickel (12, 13). It is important to note that excessive consumption of these trace elements can have detrimental effects on human health. For instance, an excessive intake of iodine has been identified as a risk factor for autoimmune thyroiditis and thyroid cancer (14, 15). Furthermore, excessive intake of nickel has been linked to the development of tumors and damage to the immune system (16, 17).

Previous studies have suggested a possible association between particular elements and gallstones development. More precisely, some researchers have found that dietary magnesium intake could decrease the risk of gallstone formation, while others have postulated that elevated blood levels of selenium may be a risk factor for gallstone formation (18, 19). After examining pertinent information, we did not discover any research examining the possible correlation between urinary iodine, nickel, and molybdenum and gallstone incidence. However, previous studies have found that excessive iodine intake may lead to hyperthyroidism and hypothyroidism, both of which promote the development of gallstones (14, 20, 21). The purpose of this study was to examine the correlation between urinary levels of iodine, molybdenum, and nickel and the occurrence of gallstones in a U.S. population and to verify whether excessive iodine intake is associated with the occurrence of gallstones.

## 2 Methods

### 2.1 Design of the study

**Figure 1:**
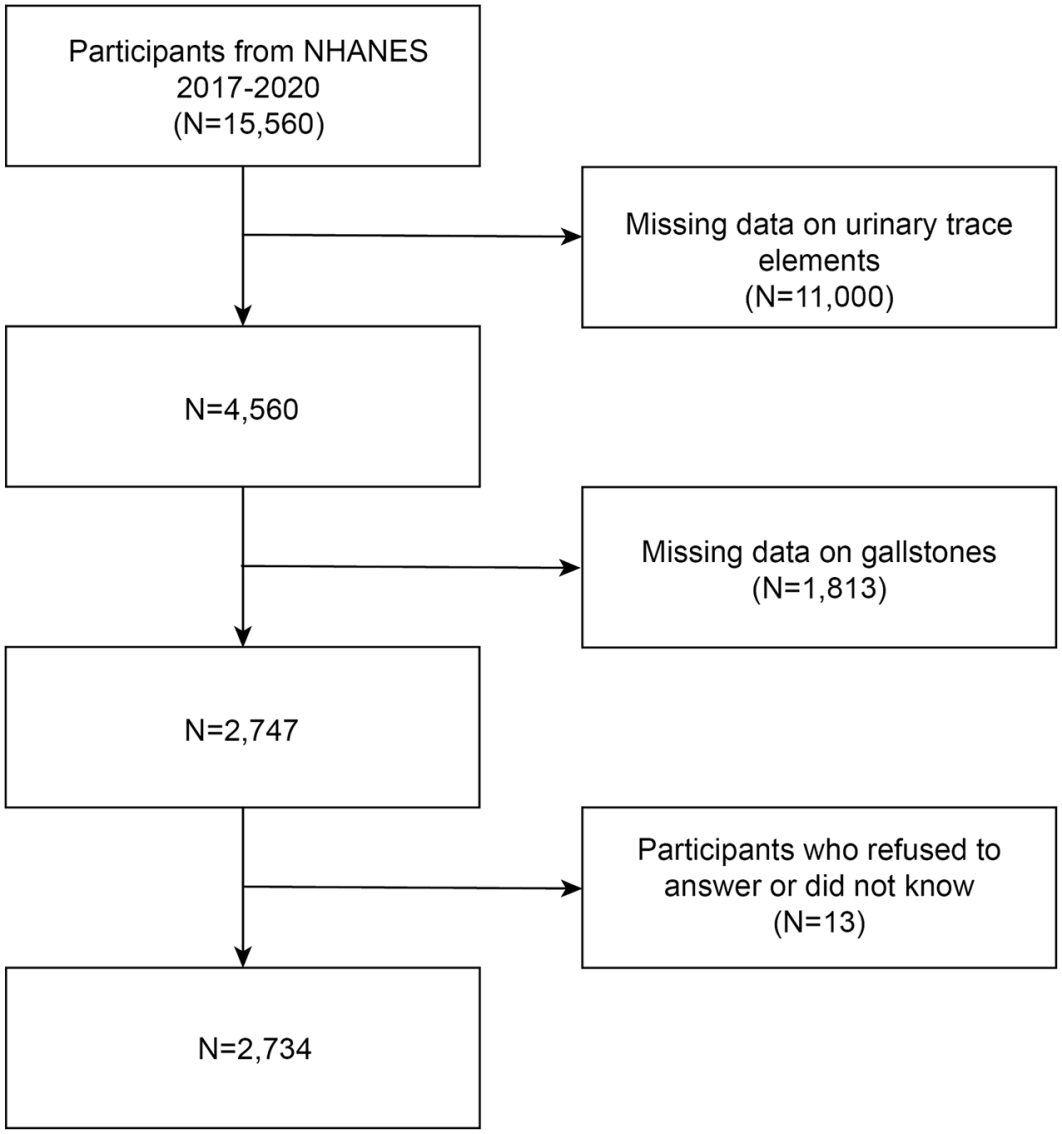
Study flowchart: NHANES enrolled a total of 15,560 participants from 2017 to 2020. Out of these individuals, 2,734 met the inclusion and exclusion criteria and were included in the analysis.

A recurrent study, the NHANES is carried out by the National Center for Health Statistics (NCHS). The purpose of this extensive national survey is to assess the nutritional and general health of Americans of all ages. The survey utilizes an extensive methodology that involves health assessments carried out at mobile health clinics, health surveys conducted at participants’ residences, as well as the extensive gathering of demographic information, medical tests, lab analyses, illnesses occur surveys, and documents of prescribed medications. This varied strategy guarantees a comprehensive assessment of multiple health-related parameters, enabling a comprehensive comprehension of the participants’ well-being. The National Center for Health Statistics’ Ethical Review Board has approved the NHANES program in its entirety, and each participant voluntarily gave informed consent. Usually, NHANES data are released every 2 years. Regretfully, the COVID-19 epidemic forced a temporary end to the program in March 2020. Consequently, a nationally representative sample that includes pre-pandemic data up until March 2020 was created by combining the data obtained between 2019 and March 2020 with the NHANES 2017–2018 cycle. Participants in this trial cycle were particularly questioned about their history of gallstones. In the beginning, the study had 15,560 participants. However, 11,000 people had missing data on urinary essential trace elements, 1,813 participants did not finish the gallstone questionnaire, and 13 participants declined to answer or did not know the questionnaires on hypertension, diabetes mellitus, and education level. The final analysis comprised 2,734 people in total, and Figure 1 presents a flow chart that details the screening procedure. The sample size formula for a cross-sectional study with qualitative data is as follow: *n* = *Z*^2^_1−α/2_ * *P* * (1−*P*)/*d*^2^, where *n* is the sample size, *Z*_1−α/2_ = 1.96 when α is taken as 0.05, *P* is the predicted prevalence of the disease, and *d* is the permissible error, which is generally taken as *d* = α/2 = 0.025 (22). When *p* = 0.1 (1), we can calculate that the minimum sample size needed for this cross-sectional study is 554. Therefore, the inclusion of 2,734 participants in our study is meeting the design requirements for a cross-sectional study.

### 2.2 How to define gallstones

In order to determine whether gallstones were present or not, we used a questionnaire called “Has your doctor ever diagnosed you with gallstones?” Gallstones were categorized as present in participants who responded positively, whereas their absence was classified in those who did not.

### 2.3 Quantification of urinary essential trace elements

Participants were asked to provide random urine by voiding into specimen cups. Urine samples were prepared, stored, and sent to the National Center for Environmental Health, Atlanta, GA, Division of Laboratory Sciences, for examination. Using inductively coupled plasma mass spectrometry (ICP-MS), the quantification of iodine (I), nickel (Ni), and molybdenum (Mo) in the urine were determined. The mass spectrometer receives liquid samples via the ICP ionization source. A nebulizer reduces the liquid samples to tiny droplets in an argon aerosol, which are subsequently injected into the ICP. Individual isotopes of an element can be identified by first allowing the ions to pass through a focusing zone, followed by the dynamic reaction cell (DRC), the quadrupole mass filter, and finally, a quick series of selective counting at the detector. For I, Mo, and Ni, the corresponding lower limits of detection (LLOD) were 2.4 μg/L, 0.80 μg/L, and 0.31 μg/L, respectively. All urinary trace element values below the limit of detection (LLOD) were substituted with the LLOD divided by √2.

### 2.4 Covariate identification

Gender, age, race, educational status, waist circumference, BMI, family income to poverty ratio, serum triglyceride concentration, and history of diabetes and hypertension were all used as covariates in the statistical model. The “Your doctor informed you that you have diabetes” questionnaire was used to ascertain if diabetes was present or not. Individuals who gave a positive response were identified as having diabetes. In a similar vein, people who answered affirmatively on the questionnaire “Your doctor informed you that you have high blood pressure” were classified as having hypertension.

### 2.5 Statistical analyses

Sample size (percentage) was used to represent categorical variables, while the mean (standard deviation) was used to express continuous variables. Two Sample *t*-test was utilized for continuous variables and chi-square tests were employed for categorical variables to evaluate differences between groups. The odds ratios (OR) and their corresponding 95% confidence intervals (CI) were determined using logistic regression to evaluate the association between every quartile of urinary levels of iodine (I), molybdenum (Mo), and nickel (Ni) with the occurrence of gallstones. Analysis involved the development of three logistic regression models. Model 1 failed to include covariate improvements, whereas Model 2 considered age, gender, and race. Model 3, after making complete adjustments, involved factors like race, age, gender, education level, family income, BMI, waist size, serum triglyceride levels, as well as the presence of hypertension and diabetes history. The relationship between the likelihood of gallstones development and urinary iodine level was examined using a restricted cubic spline analysis. Covariates such as age, gender, educational attainment, family income, waist size, BMI, and serum triglyceride concentration were taken into account while adjusting the model. Subgroup analyses were used to explore whether high levels of urinary iodine associated with a high risk of gallstones differed across subgroups of BMI, age, gender, serum triglyceride levels, diabetes, and hypertension. A random forest approach is used to fill in the missing values for the covariates. The statistical studies were conducted utilizing EmpowerStats and R 4.2.2 software.^1^

## 3 Results

### 3.1 Participants’ fundamental traits

Table 1 includes the fundamental traits and other variables of the research participants, categorized based on the existence or non-existence of gallstones. Urinary iodine, molybdenum, and nickel were grouped into quartiles based on concentration. Participants with gallstones were older, predominantly female, and of predominantly Non-Hispanic White ethnicity. In addition, they had larger BMI and waist circumference, higher rates of hypertension and diabetes, and higher serum triglyceride levels. However, there were no significant differences in HDL-C, LDL-C and Serum total cholesterol levels between participants with and without gallstones. In the quartile groups of urinary iodine concentration levels, the proportion of patients with gallstones showed a wavy increase from Quartile 1 to Quartile 4 groups. The proportion of patients with gallstones in the urinary iodine concentration Quartile 3 group was slightly lower than that in Quartile 2, while the highest proportion of participants was found in the Quartile 4 group (29.88%, *p* = 0.009). However, there was no statistically significant change in the proportion of patients with gallstones in the quartile groups of urinary molybdenum, and nickel concentration levels.

**Table 1:** Baseline characteristics of participants.

Characteristics	Total	Gallstones		*p*-value
	*n* = 2,734	Yes (*n* = 251)	No (2,483)	
Age, years, M (SD)	51 (17)	59 (16)	50 (17)	<0.001
Gender, *n* (%)				<0.001
Male	1,341 (49.0%)	73 (29.1%)	1,268 (51.1%)	
Female	1,393 (51.0%)	178 (70.9%)	1,215 (48.9%)	
Race, *n* (%)				<0.001
Mexican American	327 (12.0%)	31 (12.4%)	296 (11.9%)	
Other Hispanic	273 (10.0%)	30 (12.0%)	243 (9.8%)	
Non-Hispanic White	905 (33.1%)	111 (44.2%)	794 (32.0%)	
Non-Hispanic Black	742 (27.1%)	49 (19.5%)	693 (27.9%)	
Non-Hispanic Asian	348 (12.7%)	17 (6.8%)	331 (13.3%)	
Other Race	139 (5.1%)	13 (5.2%)	126 (5.1%)	
Education, *n* (%)				0.314
<9th grade	198 (7.2%)	12 (4.7%)	186 (7.5%)	
9–11th grade	316 (11.6%)	29 (11.6%)	287 (11.5%)	
High school graduate	695 (25.4%)	75 (29.9%)	620 (25.0%)	
Some college or AA degree	862 (31.5%)	78 (31.1%)	787 (31.6%)	
College graduate or above	663 (24.3%)	57 (22.7%)	606 (24.4%)	
BMI, M (SD)	30 (7)	32 (8)	30 (7)	<0.001
Waist circumference, cm, M (SD)	101 (17)	107 (16)	100 (17)	<0.001
Ratio of family income to poverty, M (SD)	2.59 (1.53)	2.60 (1.43)	2.59 (1.54)	0.932
Serum triglyceride, mg/dL, M (SD)	113 (88)	122 (53)	115 (92)	0.043
HDL-C, mg/dL, M (SD)	54 (15)	54 (14)	54 (16)	0.808
LDL-C, mg/dL, M (SD)	109 (31)	110 (24)	111 (26)	0.432
Serum total cholesterol, mg/dL, M (SD)	185 (39)	185 (38)	185 (39)	0.936
I, ug/L, *n* (%)				0.009
Q1 [2.6, 70.8]	684 (25.0%)	41 (16.3%)	643 (25.9%)	
Q2 [70.8, 124]	681 (24.9%)	68 (27.1%)	613 (24.7%)	
Q3 [124, 230]	684 (25.0%)	67 (26.7%)	617 (24.8%)	
Q4 [230, 23637.3]	685 (25.1%)	75 (29.9%)	610 (24.6%)	
Mo, ug/L, *n* (%)				0.268
Q1 [0.93, 18.8]	684 (25.0%)	74 (29.5%)	610 (24.5%)	
Q2 [18.8, 36.5]	683 (25.0%)	61 (24.3%)	622 (25.1%)	
Q3 [36.5, 63.2]	683 (25.0%)	53 (21.1%)	630 (25.4%)	
Q4 [63.2, 665]	684 (25.0%)	63 (25.1%)	621 (25.0%)	
Ni, ug/L, *n* (%)				0.545
Q1 [0.22, 0.65]	671 (24.6%)	56 (22.3%)	615 (24.8%)	
Q2 [0.65, 1.17]	687 (25.1%)	68 (27.1%)	619 (24.9%)	
Q3 [1.17, 1.95]	690 (25.2%)	58 (23.1%)	632 (25.5%)	
Q4 [1.95, 64.1]	686 (25.1%)	69 (27.5%)	617 (24.8%)	
Rate of hypertension, *n* (%)	1,069 (39.1%)	126 (50.2%)	943 (38.0%)	<0.001
Rate of diabetes, *n* (%)	496 (18.1%)	67 (26.7)%	429 (17.2%)	<0.001

M, mean values; SD, standard deviation; *n*, sample size; BMI, body mass index; HDL-C, high-density lipoprotein cholesterol; LDL-C, low-density lipoproteins cholesterol; I, iodine; Mo, molybdenun; Ni, nikel.

### 3.2 Relationship between the risk of gallstones and urinary essential trace elements

**Figure 2:**
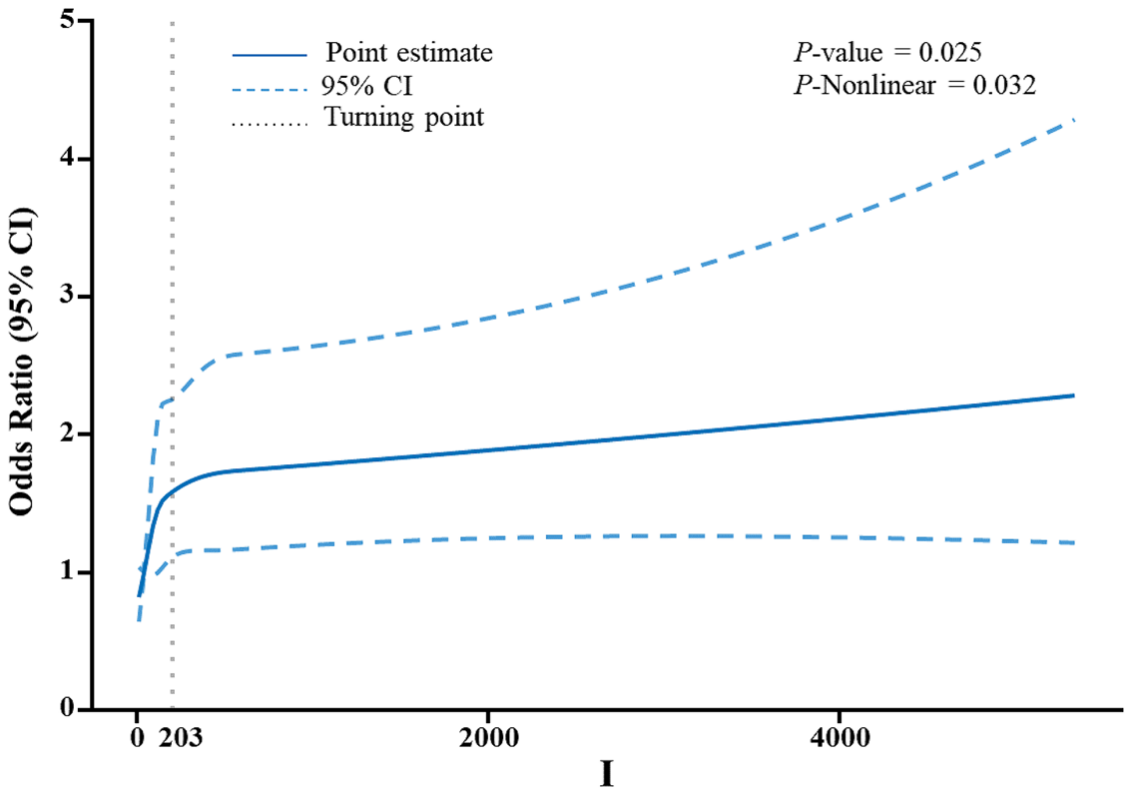
An analysis utilizing a restricted cubic spline was conducted to evaluate the correlation between levels of iodine in urine and the risk of developing gallstones. The adjustment of the model included factors such as age, gender, educational attainment, household income, body mass index (BMI), waist circumference, and serum triglyceride levels. The depiction of odds ratios (OR) was presented as a continuous solid blue line, with the incorporation of 95% confidence intervals displayed as blue dashed lines. Furthermore, the inflection point was visually indicated by gray dotted lines.

The relationship between the risk of gallstones development and urinary essential trace elements is shown in Table 2. Participants were grouped into quartiles based on the levels of iodine, nickel, and molybdenum in their urine for analysis. The control group was defined as the quartile with the lowest concentration of each element. Urinary iodine levels ranged from 2.6ug/L to 70.8ug/L in Quartile 1, from 70.8ug/L to 124ug/L in Quartile 2, from 124ug/L to 230ug/L in Quartile 3 and from 230ug/L to 23637.3ug/L in Quartile 4. In the first model, which was the initial model without adjusting for covariates, the likelihood of developing gallstones was found to be linked to urinary iodine levels. Participants in Quartiles 2–4 showed a 73, 70, and 93% increased risk of getting gallstones, respectively, in comparison to those in Quartiles 1. Within the second model, those in Quartiles 2–4 had a 76, 74, and 75% greater risk of getting gallstones, respectively, in comparison to those in Quartile 1, after controlling for gender, age, and race. In adjusted model 3 controlling for gender, age, race, education, household income, BMI, waist size, Serum triglyceride concentration, as well as previous diagnoses of diabetes and high blood pressure, an elevated level of urinary iodine was correlated with a higher risk of getting gallstones. Individuals in Quartiles 2–4 displayed a 69, 68, and 65% rise in gallstone risk, respectively, in comparison to those in Quartile 1. Neither Model 1, Model 2, nor Model 3 showed any correlation between the risk of gallstones and urinary nickel and molybdenum levels. Furthermore, restricted cubic spline analysis revealed what Figure 2 showed that urinary iodine levels were nonlinearly positively correlated with the development of gallstones (*P*-Nonlinear = 0.032). When the urinary iodine concentration was less than 203 ug/L, the curve of OR was steeper than the curve when the urinary iodine concentration was greater than 203 ug/L.

**Table 2:** Association between trace elements and gallstones.

	OR (95% CI), *p*-value		
	Model 1	Model 2	Model 3
I, ug/L			
Q1 [2.6, 70.8]	1.00 (reference)	1.00 (reference)	1.00 (reference)
Q2 [70.8, 124]	1.73 (1.16, 2.61), 0.007	1.76 (1.17, 2.68), 0.007	1.69 (1.11, 2.56), 0.014
Q3 [124, 230]	1.70 (1.14, 2.57), 0.010	1.74 (1.15, 2.65), 0.009	1.68 (1.10, 2.55), 0.016
Q4 [230, 23637.3]	1.93 (1.30, 2.88), 0.001	1.75 (1.16, 2.66), 0.008	1.65 (1.09, 2.51), 0.018
Mo, ug/L			
Q1 [0.93, 18.8]	1.00 (reference)	1.00 (reference)	1.00 (reference)
Q2 [18.8, 36.5]	0.81 (0.56, 1.15), 0.239	0.84 (0.58, 1.21), 0.342	0.78 (0.53, 1.13), 0.189
Q3 [36.5, 63.2]	0.69 (0.48, 1.00), 0.051	0.80 (0.55, 1.17), 0.258	0.73 (0.49, 1.08), 0.118
Q4 [63.2, 665]	0.83 (0.58, 1.19), 0.318	1.10 (0.76, 1.59), 0.601	1.00 (0.69, 1.46), 0.987
Ni, ug/L			
Q1 [0.22, 0.65]	1.00 (reference)	1.00 (reference)	1.00 (reference)
Q2 [0.65, 1.17]	1.21 (0.83, 1.75), 0.321	1.25 (0.85, 1.83), 0.257	1.14 (0.77, 1.68), 0.521
Q3 [1.17, 1.95]	1.01 (0.69, 1.48), 0.968	1.11 (0.75, 1.65), 0.610	1.04 (0.70, 1.56), 0.838
Q4 [1.95, 64.1]	1.23 (0.85, 1.79), 0.272	1.26 (0.86, 1.86), 0.229	1.17 (0.80, 1.74), 0.418

Model 1: no adjustment for covariates.

Model 2: covariates such as gender, age, and race were included.

Model 3: a comprehensive model incorporating age, gender, race, educational level, family income, BMI, waist circumference, Serum triglyceride level, and a history of diabetes and hypertension.

### 3.3 Subgroup analyses

**Figure 3:**
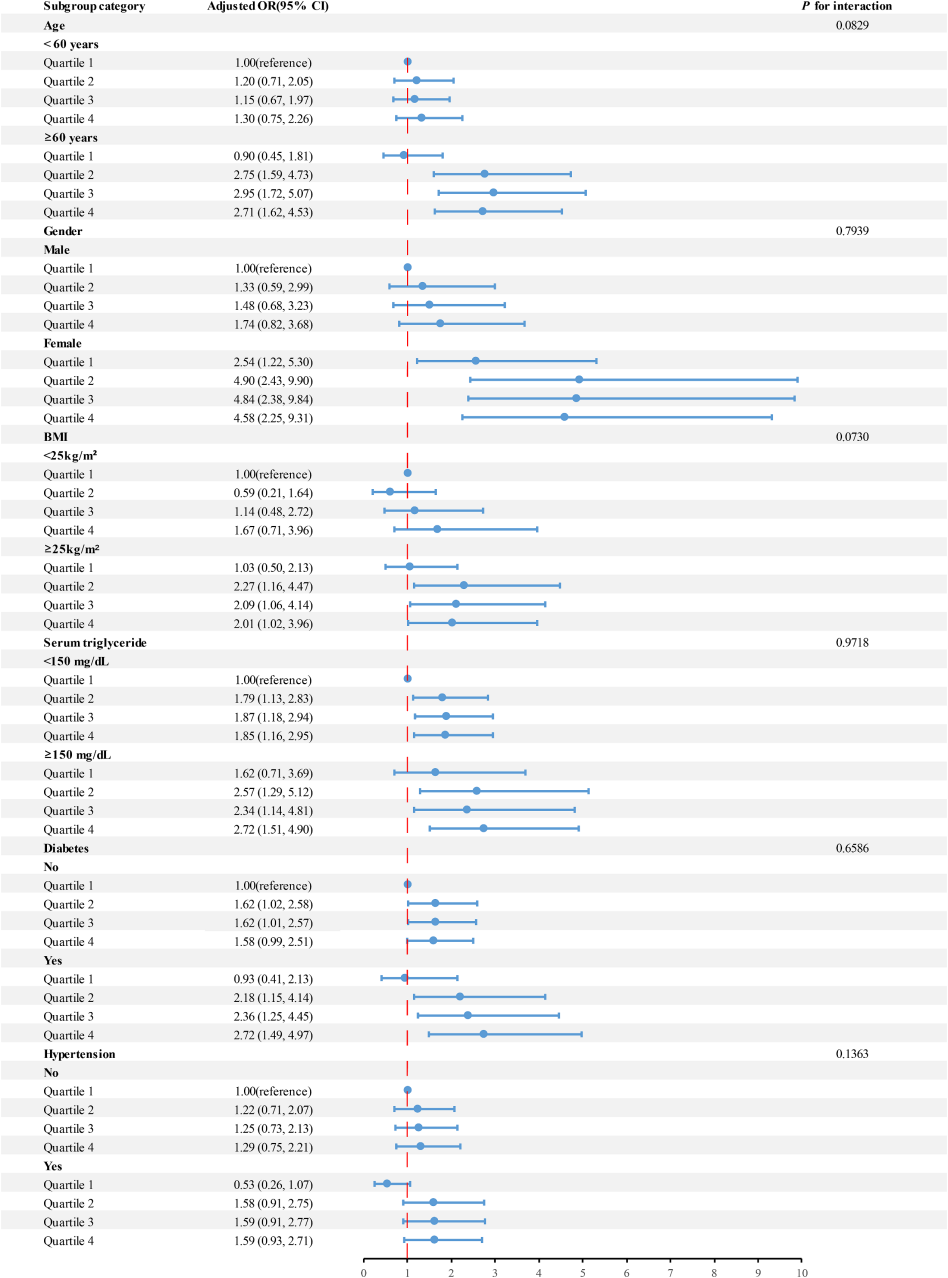
Analyses were performed on subgroups to explore if various factors such as age, gender, BMI, serum triglyceride levels, hypertension, and diabetes influenced the connection between urinary iodine levels and the risk of developing gallstones.

Studies were performed on subgroups to explore if various factors such as gender, age, BMI, serum triglyceride concentration, diabetes, and hypertension influenced the connection between urinary iodine levels and the likelihood of gallstone formation (Figure 3). Upon controlling for pertinent variables, our study revealed variations in participants based on age, gender, BMI, and diabetes status. Notably, Quartiles 2–4 of urinary iodine concentration exhibited a strong correlation with the risk of developing gallstones among individuals aged 60 and above, female, had a BMI of 25 or higher, and were diagnosed with diabetes.

## 4 Discussion

Using information from the 2017–2020 NHANES, this cross-sectional study looked into any possible relationship between the occurrence of gallstones and essential trace elements in urine. Even after controlling for relevant variables, the results point to a substantial correlation between higher urinary iodine concentration and an elevated risk of gallstones. Notably, those 60 years of age or older, women, those with a BMI of 25 or above, and those with a diagnosis of diabetes showed the strongest correlation with this association. However, elevated levels of nickel, and molybdenum in the urine were not associated with an increased risk of gallstones.

To the best of our knowledge, there have been no previous studies specifically examining the correlation between urinary trace elements levels and gallstones, but our results indicate that urinary iodine levels are positively associated with the risk of getting gallstones, whereas elevated levels of nickel, and molybdenum are not. In the context of China, Mei-Hsuan Lee et al. collected data on serum metal levels in a study covering 701 patients with gallstones and concluded that serum levels of cadmium, chromium, copper, molybdenum, and vanadium were associated with the development of gallstones (23). Additionally, blood selenium was reported by Wang et al. as a separate risk indicator for gallstones in the USA using data from the NHANES database (18). Currently, no definitive conclusions have been drawn regarding the effects of trace elements (both metallic and nonmetallic) on the occurrence of gallstones in humans. Therefore, it is noteworthy that our research indicates a link between increased iodine levels in urine and a higher risk of gallstone development.

Iodine is an important trace element that is closely related to human growth, development and metabolism (24). Iodine is mainly consumed through the diet and is rapidly and almost completely absorbed in the stomach and duodenum (>90%). More than 90% of the iodine ingested is ultimately excreted in the urine, with the remaining portion excreted in the feces or sweat (5–10%). Urinary iodine concentration is therefore a sensitive indicator of recent iodine intake (25–28). Iodine deficiency is usually caused by a lack of iodine in the diet and is particularly common in inland areas. Iodine deficiency is the main cause of diseases such as stunting, developmental delay, and endemic goiter (29). For this reason, in 1994, the WHO proposed a global strategy to add potassium iodate to salt to ensure adequate iodine in the diet. This strategy to control iodine deficiency has yielded tremendous results, with approximately 70% of households worldwide having access to adequately iodized salt. However, in order to ensure adequate iodine intake, iodine overdose should also be avoided, and data from the WHO show that adequate or excessive iodine intake has been observed in many countries (30, 31). In addition to the overuse of salt iodization, there are other factors that may contribute to excessive iodine intake. For example, the daily consumption of certain iodine-rich foods such as seaweeds (32), and the use of iodine-containing water purification tablets lead to excessive iodine in drinking water (33), in addition to the medical use of iodine-containing medications such as amiodarone and iodine-containing contrast media, which are also important contributors to excessive iodine intake (34, 35).

Although there have been no definitive experimental studies linking excessive iodine intake to the development of gallstones, previous studies have found that excessive iodine intake may lead to a variety of disorders, including hypothyroidism, hyperthyroidism, and autoimmune thyroid disease (ATD) (14). Hypothyroidism and hyperthyroidism may play an important role in the association between excessive iodine intake and the development of gallstones. Kube I et al. found that hypothyroidism increases the hydrophobicity of primary bile acids and thus increases the incidence of gallstones (36). Wang Y et al. found that both hypothyroidism and hyperthyroidism can contribute to the development of gallstones through different mechanisms (20). Nakano S et al. concluded that rapid weight loss due to hyperthyroidism can lead to the development of gallstones (37). In addition, high levels of iodine intake have effects on blood glucose, blood pressure and lipid metabolism (38, 39), and may contribute to the development of gallstones (1, 9). The main components of bile include bile acids, cholesterol and phospholipids, which are excreted in certain proportions. When a component is oversaturated, stones are formed, the most common of which are cholesterol stones (1). CYP7A1, also known as cholesterol 7α-hydroxylase, is the key enzyme that determines the rate at which cholesterol is converted to bile acids. Thyroid hormone enhances the expression of CYP7A1 mRNA, which is essential for the role of thyroid hormone in regulating cholesterol levels (40, 41). In hypothyroidism, cholesterol levels are elevated, which ultimately leads to the formation of gallstones. In addition, in hypothyroidism, the number of LDL receptors in the liver decreases, resulting in the failure to remove some of the LDL, a decrease in bile flow and the development of sphincter of Oddi disfunction, which ultimately leads to the formation of gallstones as well (20). Hepatic secretion of bile salts, phosphatidylcholine and cholesterol is mainly determined by ATP-binding cassette (ABC) transporters on the apical membrane of hepatocytes (20, 42). Multiple receptors, namely the intrahepatocytic nuclear retinoid X receptor (RXR), and liver X receptor (LXR), have been discovered to govern the regulation of these ABC transporters. Hyperthyroidism is induced by up-regulation of the expression of the liver nuclear receptor genes LXRα and RXR expression induces cholesterol gallstone formation (20). Therefore, we believe that hypothyroidism and hyperthyroidism due to excessive iodine intake are the main causes of gallstone formation.

Subgroup analyses showed differences in urinary iodine levels and gallstone risk in specific populations. Obesity is an important risk factor for the development of gallstones, mainly because obesity leads to abnormalities including hepatic and dyslipidemia, which are manifested by excessive bile secretion by the liver, as well as the development of hyperlipidemia. In addition, obesity leads to insulin resistance, which has been shown to be associated with gallbladder stone formation (43, 44). Estrogen increases the risk of gallstone formation by stimulating the hepatic synthesis and secretion of cholesterol while blocking the synthesis of bile salts. In addition, the elderly and diabetes are also recognized as independent risk factors for gallstone formation (1, 8, 44, 45). This may explain the fact that the correlation between high urinary iodine levels and a high risk of gallstones is particularly pronounced in people aged 60 and older, women, had a BMI of 25 or more, and those diagnosed with diabetes mellitus.

Restricted cubic spline analysis showed that urinary iodine levels were nonlinearly positively correlated with the occurrence of gallstones (*P*-Nonlinear = 0.032). When the urinary iodine concentration was less than 203 ug/L, the curve of OR was steeper than the curve when the urinary iodine concentration was greater than 203 ug/L. Unfortunately we did not find a mechanism that causes this phenomenon, and the relevant mechanisms need to be further investigated.

The following are the strengths of this study. First off, the study offers valuable guidance for avoiding and decreasing the incidence of gallstones in Americans. Second, the study’s data came from NHANES, which employed a standardized experimental testing technique and a nationally representative sample to successfully minimize study mistakes. This study has various restrictions. First, the diagnosis of gallstones was obtained through a self-report questionnaire, which may have led to inaccurate diagnoses and errors in participant recall. Second, iodine levels in urine were obtained from a single sample and were not sampled multiple times over a period of time, which does not reflect participants’ long-term iodine intake levels. In addition, in this cross-sectional study, a large number of participants were not tested for iodine levels in urine and did not provide self-reports of gallstones, resulting in a reduced number of participants and reducing the validity of the experimental results.

To summarize, our research revealed a correlation between high levels of iodine in the urine and a heightened risk of developing gallstones. It is worth mentioning that this connection was especially noticeable in individuals aged 60 and above, females, individuals with a body mass index of 25 or greater, and those diagnosed with diabetes. Urinary iodine levels serve as indicators of the body’s iodine status, thus suggesting that excessive iodine intake may be linked to an elevated risk of gallstone formation. There is no correlation between elevated urinary levels of nickel and molybdenum and a higher risk of gallstones. In the next step of our research, we will use cohort studies, case–control studies, and animal experiments to discover the causal relationship between urinary iodine levels and the occurrence of gallstones. These studies will deepen our understanding of iodine intake and the etiology of gallstone disease and help us to properly use iodine supplements and prevent gallstones.

## Data availability statement

Publicly available datasets were analyzed in this study. This data can be found at: https://www.cdc.gov/nchs/nhanes/index.htm.

## Ethics statement

The studies involving humans were approved by the Ethical Review Board of the National Center for Health Statistics. The studies were conducted in accordance with the local legislation and institutional requirements. The participants provided their written informed consent to participate in this study.

## Author contributions

YL: Conceptualization, Investigation, Software, Writing – original draft, Writing – review & editing, Formal analysis, Methodology. MW: Data curation, Formal analysis, Investigation, Writing – original draft. WD: Conceptualization, Data curation, Methodology, Writing – original draft. LQ: Data curation, Formal analysis, Supervision, Writing – review & editing. XL: Data curation, Formal analysis, Writing – review & editing. XF: Conceptualization, Data curation, Formal analysis, Funding acquisition, Investigation, Project administration, Resources, Validation, Writing – original draft, Writing – review & editing.

## Footnotes

^1^https://www.empowerstats.com
